# The existence of fertile hybrids of closely related model earthworm species, *Eisenia andrei* and *E*. *fetida*

**DOI:** 10.1371/journal.pone.0191711

**Published:** 2018-01-25

**Authors:** Barbara Plytycz, Janusz Bigaj, Artur Osikowski, Sebastian Hofman, Andrzej Falniowski, Tomasz Panz, Pawel Grzmil, Franck Vandenbulcke

**Affiliations:** 1 Department of Evolutionary Immunology, Institute of Zoology and Biomedical Research, Jagiellonian University, Krakow, Poland; 2 Department of Animal Anatomy, University of Agriculture in Krakow, Krakow, Poland; 3 Department of Comparative Anatomy, Institute of Zoology and Biomedical Research, Jagiellonian University, Krakow, Poland; 4 Department of Malacology, Institute of Zoology and Biomedical Research, Jagiellonian University, Krakow, Poland; 5 Faculty of Biochemistry, Biophysics and Biotechnology, Jagiellonian University, Krakow, Poland; 6 Department of Genetics and Evolution, Institute of Zoology and Biomedical Research, Jagiellonian University, Krakow, Poland; 7 Ecologie Numerique et Ecotoxicologie, University Lille Nord de France, Lille, France; Imperial College London, UNITED KINGDOM

## Abstract

Lumbricid earthworms *Eisenia andrei* (Ea) and *E*. *fetida* (Ef) are simultaneous hermaphrodites with reciprocal insemination capable of self-fertilization while the existence of hybridization of these two species was still debatable. During the present investigation fertile hybrids of Ea and Ef were detected. Virgin specimens of Ea and Ef were laboratory crossed (Ea+Ef) and their progeny was doubly identified. 1 –identified by species-specific maternally derived haploid mitochondrial DNA sequences of the COI gene being either ‘a’ for worms hatched from Ea ova or ‘f’ for worms hatched from Ef ova. 2 –identified by the diploid maternal/paternal nuclear DNA sequences of 28s rRNA gene being either ‘AA’ for Ea, ‘FF’ for Ef, or AF/FA for their hybrids derived either from the ‘aA’ or ‘fF’ ova, respectively. Among offspring of Ea+Ef pairs in F1 generation there were mainly aAA and fFF earthworms resulted from the facilitated self-fertilization and some aAF hybrids from aA ova but none fFA hybrids from fF ova. In F2 generation resulting from aAF hybrids mated with aAA a new generations of aAA and aAF hybrids were noticed, while aAF hybrids mated with fFF gave fFF and both aAF and fFA hybrids. Hybrids intercrossed together produced plenty of cocoons but no hatchlings independently whether aAF+aAF or aAF+fFA were mated. These results indicated that Ea and Ef species, easy to maintain in laboratory and commonly used as convenient models in biomedicine and ecotoxicology, may also serve in studies on molecular basis of interspecific barriers and mechanisms of introgression and speciation. Hypothetically, their asymmetrical hybridization can be modified by some external factors.

## Introduction

The composting earthworms belonging to the genus *Eisenia* (Annelida, Oligochaeta, Lumbricidae) are easy to maintain under laboratory conditions and exhibit a relatively simple body organization. *E*. *andrei* and *E*. *fetida* are explicitly required by the international Guidelines [1, 2, 3] as test species in ecotoxicology (e.g. [4–7]), and are widely used as models for basic studies in vermicomposting (e.g. [8, 9]), and biomedicine [10–12]. Because the molecular-genetic platforms used in these studies are both sensitive and especially specific, it is crucial that the two eco-physiologically similar commonly used species, *Eisenia andrei* (*Ea*; ‘red worms’) and *E*. *fetida* (*Ef*; ‘tiger worms’ or ‘brandlings’) are reliably identified. Originally these two species were considered as pigmentation morphs of *E*. *foetida*, later as its subspecies. Currently they are recognized as two distinct species possessing specific sequences of mitochondrial DNA and nuclear DNA sequences [13–17]. Reproductive isolation in laboratory experiments [18, 19] allowed identification of differences at molecular level (e.g. [20]) including species-specific fluorophore fingerprints [21]. Despite these differences, some data (e.g. [22]) including findings from our own pilot studies showing that these two species can potentially inter-breed to yield hybrids and fertile hybrid offspring. This calls for special care to avoid potential source of error and misinterpretation of data collected during work with specimens of unclear genetic origin.

During our investigation concerning immunity, regenerative abilities and ecotoxicology of some lumbricid worms we have developed several reliable markers allowing distinction between Ea and Ef worms [23, 24]. However, some pieces of evidence, such as the intermediate pigmentation patters and the presence of the MUG-like fluorophore considered to be specific for Ea earthworms in several Ef-like specimens delimitated as Ef by the mitochondrial COI gene [12, 23], suggest that hybrids exist. Like in most animals, haploid mitochondrial (mt) genes of earthworms are uniparentally inherited from the ova, thus maternal origin [25]. Consequently, an individual is either ‘mt-a’ or ‘mt-f’. Species-specific COI sequences are thus useless for detection of hermaphroditic individuals [26]. Detection of hermaphroditic individuals can be done on the basis of the diploid nuclear (nu) sequences, e.g. 28S rRNA genes [13] that can be either nu-AA (in *E*. *andrei*), nu-FF (in *E*. *fetida*), or nu-AF (in hybrids).

Although most members of the Lumbricidae earthworm family are cross-fertilizing hermaphrodites with reciprocal insemination [27, 28], self-fertilization has also been recorded in *Eisenia* species [29]. This introduces a potential genetic-background complication in laboratory studies of Ea+Ef experimental pairings where the F1 offspring could be expected to be either hybrids EaxEf or self-fertilized Ea or Ef specimens.

The aim of this work was to test two hypotheses. 1—genotypically-defined Ea and Ef earthworms can produce both self-fertilized specimens and hybrid offspring when intercrossed (Ea+Ef). 2—some of the hybrids are fertile. The results evidenced the existence of fertile hybrids between *E*. *andrei* and *E*. *fetida* from laboratory stocks reared for generations in Lille/Krakow.

## Materials and methods

### Experimental animals

Adult composting earthworms *E*. *andrei* (Ea) and *E*. *fetida* (Ef) were delivered from laboratory colonies maintained at Lille University (France) and then reared for generations in the Institute of Zoology and Biomedical Research, Jagiellonian University in Krakow (Poland). In Krakow laboratory, worms of both species were kept separately in plastic boxes with perforated lids in soil from a commercial supplier (PPUH BIOVITA, Tenczynek, Poland) at room temperature and natural illumination, and fed ad libitum on mixed diet composed of boiled/dried/powdered tea, nettle and dandelion leaves.

From the stock colonies previously genotyped in Krakow [23], groups of typical representatives of both species, i.e. 16 uniformly red specimens (Ea), and 16 striped specimens (Ef), were selected for present investigations and reared separately under the same conditions.

### Experimental design

The aim of proof-of-concept preliminary experiments was to compare the cocoon production and hatchability in the laboratory formed intra-specific pairs Ea+Ea and Ef+Ef, and inter-specific pairs (Ea+Ef).

**Fig 1:**
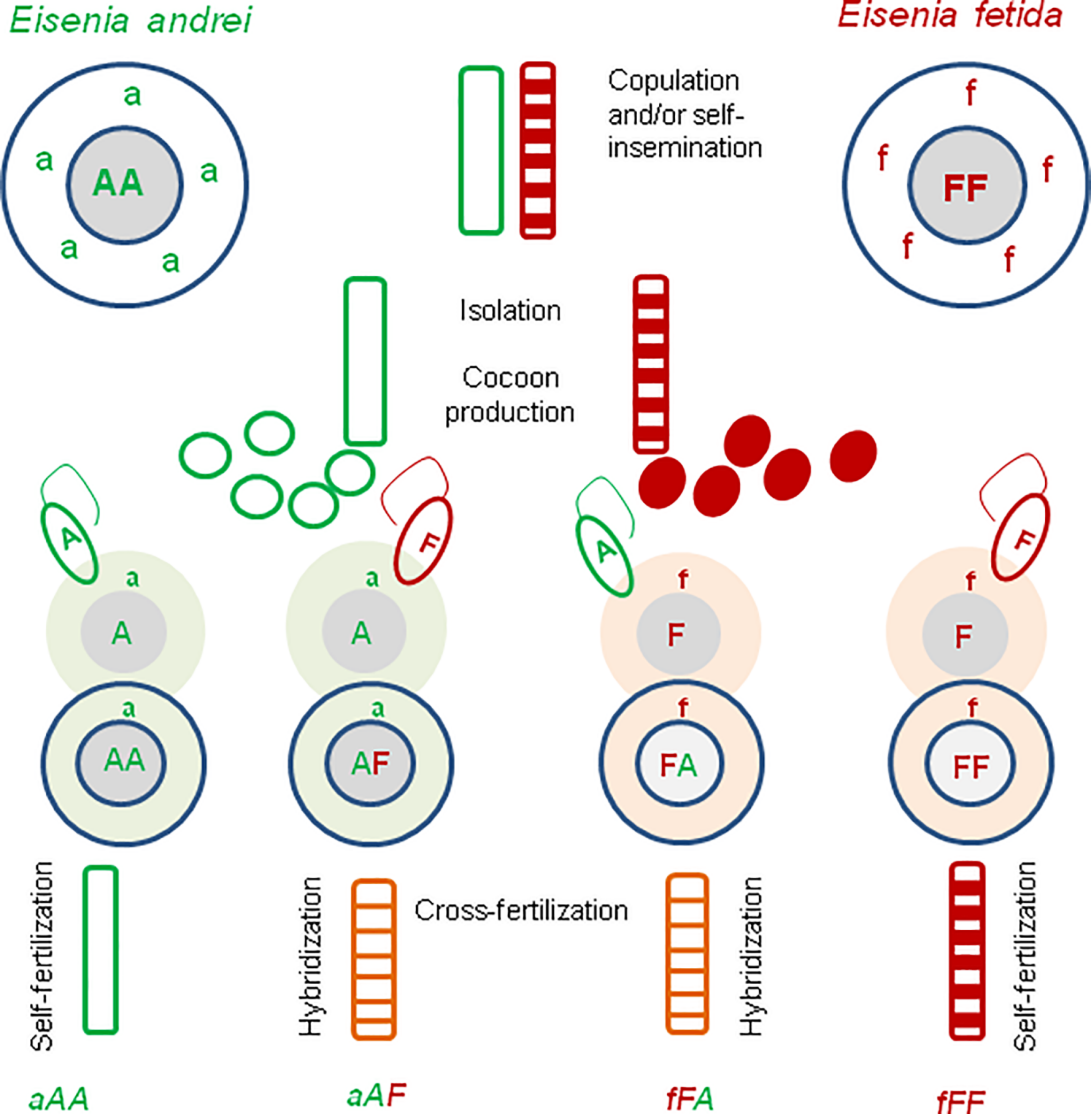
Scheme of mating experiments and hybrid identification. Composting earthworms Ea and Ef are cross-fertilizing simultaneous hermaphrodites capable also to self-fertilization [29]. A few days after copulation and/or self-insemination each specimen produces cocoons containing its own eggs that are fertilized within cocoon by spermatozoa stored previously in spermatheca. Ova might be fertilized either by spermatozoa of the same specimen or cross-fertilized by spermatozoa coming from the partner.

Since viable hatchlings were obtained in all three groups of experimental couples of earthworms, a possibility of hybridization versus self-fertilization in Ea+Ef pairs of earthworms was tested. Analyzes of species-specific sequences of the haploid mitochondrial marker COI (marked by letters ‘a’ or ‘f’) showing maternity and of the maternal/paternal nuclear marker 28S rRNA gene (marked by capital letters ‘A’ or ‘F’) were performed. Self-fertilized specimens of Ea and Ef must exhibit aAA or fFF markers, respectively, while hybrids of Ea+Ef shall possess two easily identified AF/FA sequences, thus they shall be either aAF or fFA, with the first nuclear marker consistent with maternity (Fig 1).

The DNA samples were obtained from the tail tips of earthworms from the controlled laboratory mating of Ea+Ef pairs and their progeny (potentially aAA, fFF, aAF, or fFA). Photos were also performed to investigate inheritance of pigmentation patterns.

### Experimental scheme

#### Proof-of-concept preliminary experiments

To avoid copulation and uncontrolled sperm exchange, very small (<0.05 g b.w.) freshly hatched specimens were collected from Ea and Ef colonies of genotyped earthworms. On December 15, a culture of 15 pairs of virgin worms was started till sexual maturation and reproduction, forming the control intra-specific couples, 4 (Ea+Ea) and 4 (Ef+Ef), and 7 experimental inter-specific (Ea+Ef) pairs. Progeny of intra-specific mating served as a source of individuals of pure aAA and fFF genotypes for further studies, while several generations of progeny of inter-specific couples were used for detection of hybrid specimens.

Eight weeks into the experiment, both the control pairs and experimental worms were transferred to new soil and their cocoons produced over the two consecutive weeks (9^th^ and 10^th^) were counted and kept in separate boxes for monitoring their viability/hatchability during the 5 consecutive weeks. Hatchlings were used for further mating.

#### Progeny of (Ea+Ef) couples

Freshly hatched offspring of the (Ea+Ef) couples were associated in pairs, each in a separate box. Pairs of virgin earthworms were joined randomly since identity of such neonate earthworms is impossible to establish by the phenotype. This distribution hypothetically led to the formation of either intra-specific couples (aAA+aAA; fFF+fFF), or hybrid-hybrid couples (aAF+fFA; aAF+aAF; fFA+fFA), or back-crosses of hybrids with Ea or Ef (aAF+aAA or aAF+fFF; fFA+aAA or fFA+fFF). After several weeks, the pairs produced cocoons, some of which were viable, and the procedure was repeated. In order to prevent stress in the juvenile earthworms, which could be potentially disruptive factor affecting their maturation/reproduction, the genetic identification of each particular earthworm was established only during its adulthood, when cocoon production was well established (at body weight >0.5 g).

For the latter purpose, some adults (with special attention to those exhibiting atypical pigmentation patterns), were settled individually in boxes on wet filter papers. After overnight depuration, their 4–6 posterior segments were amputated and ethanol-fixed for DNA analysis while earthworms returned to soil for further reproduction. Tail tips of *Eisenia* sp. easily regenerate [30] thus–when necessary–tails were amputated again from the same specimens.

Pigmentation patterns were documented by photography with the DSL camera (Sony SLT-A58).

### DNA extraction, PCR amplification and sequencing

The terminal segments of the earthworms were fixed in 80% ethanol. The tissues were hydrated in Tris-EDTA (TE) buffer (3×10 min); then, total genomic DNA was extracted using the SHERLOCK^®^ extracting kit (A&A Biotechnology). The final product of purification was resuspended in 20μl of TE buffer. The extracted DNA was then stored at − 80°C.

Two molecular markers were used: cytochrome oxidase subunit I (COI) for the mitochondrial DNA (mtDNA) and the 28S subunit of the nuclear ribosomal RNA (28S) for the nuclear DNA (ncDNA).

For COI the following primers were used:

LCOI490 5 ′ - GGTCAACAAATCATAAAGATATTGG—3 ′ [31] and COR722b 5 ′ - TAAACTTCAGGGTGACCAAAAAATYA—3 ′ [32]. Amplifications were performed in 50 μl reaction mixtures containing approximately 20 ng of genomic DNA, 5 μl of 10x Taq Reaction Buffer, 5 μl of 25 mM MgCl_2_, 200 μM of each dNTP, 0,8 μg of BSA, 2,5 μl of 10% TWEEN, 1,25 μM of both the forward and reverse primers and 0,1 U/μl of Taq polymerase (Thermo Scientific). The PCR conditions were as follows: for COI: initial denaturation step of 4 min at 94°C, followed by 35 cycles of 1 min at 94°C, 1 min at 55°C, 2 min at 72°C, and a final extension of 4 min at 72°C.

The primers used for 28S amplification were designed based on sequences available in GenBank for the genus *Eisenia* [13]: 28S-DZDZ-F 5 ′ - AATAGCCCAGCACCGAATC—3 ′ and 28S-DZDZ-R 5 ′ - ACTCCTTGGTCCGTGTTTC—3 ′. For 28S amplification, the Finnzymes Phusion^®^ High-Fidelity DNA Polymerase (ThermoFisher Scientific) was used. Amplifications were performed in 20 μl reaction mixtures containing ~100 ng of genomic DNA, 4 μl of the 5× Phusion GC buffer, 200 μM of each dNTP, 1 μM of both the forward and reverse primers and 0.02 U/μl of Phusion DNA polymerase. The PCR cycling conditions were as follows: a first denaturation at 98°C for 60 s; 36 cycles of 98°C for 10 s, 54°C for 20 s and 72°C for 20 s; and a final extension of 5 min at 72°C.

To check the quality of the PCR products, 4 μl of reaction product were run on 1% agarose gel. PCR products were purified using Clean-Up^®^ columns (A&A Biotechnology). The purified PCR products were sequenced in both directions using BigDye Terminator v3.1 Cycle Sequencing Kit (Applied Biosystems) following the manufacturer’s protocol and using the primers described above. After removing the terminators with ExTerminator^®^ columns (A&A Biotechnology) the sequencing reaction products were separated on an ABI PRISM®3100 Avant Genetic Analyzer in commercial laboratory Genomed S.A. (Warszawa, Poland).

All the sequences were declared in the GenBank (MG030809—MG030998 for 28S and MG030999—MG031156 for COI).

## Results

### Results of proof-of-concept preliminary experiments

Features of cocoons produced during the 9^th^ and the 10^th^ weeks by 15 pairs of earthworms put to boxes as pairs of neonate virgin specimens, i.e. 4(Ea+Ea), 4(Ef+Ef), and 7(Ea+Ef), were followed for further 5 weeks and the results are shown in Table 1.

**Table 1:** Reproductive success of earthworms kept as intra-specific pairs of *E*. *andrei* (Ea+Ea) and *E*. *fetida* (Ef+Ef), or inter-specific pairs (Ea+Ef) measured by viability and hatchability of cocoons laid during the 9^th^ and 10^th^ weeks after joining of pairs of small virgin specimens.

Reproduction	4 pairs (Ea+Ea)	4 pairs (Ef+Ef)	7 pairs (Ea+Ef)
Pairs with cocoons	4/4	4/4	7/7
Numbers of cocoons per earthworm per week (X+SE)	2.9+0.43	3.4+0.68	4.3+0.42
Numbers of hatchlings per earthworm per week (X+SE)	5.6+0.39	2.8+1.05	1.0+0.64
Numbers of pairs without offspring/total number of pairs	0/4	0/4	3/7
Sterile cocoons (%)	7	48	77

Numbers of cocoons laid by one worm per week were similar in all groups of worms, being lowest in Ea+Ea (2.9+0.43), medium in Ef+Ef (3.4+0.68) and highest in boxes with inter-specific couples Ea+Ef (4.3+0.42). However, numbers of hatchlings per earthworm per week were in opposite sequence, being highest in Ea boxes (5.6+0.39), medium in Ef (2.8+1.05) and lowest in (Ea+Ef) boxes (1.0+0.64). Moreover, hatchlings were present in each of four Ea and four Ef boxes, while 3 of 7 boxes with inter-species pairs contained only sterile cocoons, without any hatchlings. The percentages of sterile cocoons was low (7%) in (Ea+Ea), quite high (48%) in (Ef+Ef), but very high (77%) in (Ea+Ef) groups.

In general, reproductive output was most efficient in *E*. *andrei*, lower in *E*. *fetida*, whilst it was very low in inter-specific pairings (Table 1). For present purposes it was important to check whether hatchlings from partners of inter-specific pair have resulted from self-fertilization or cross-fertilization (hybridization).

### Earthworm genotyping and genealogy considerations

#### Sequence characterization

**Fig 2:**
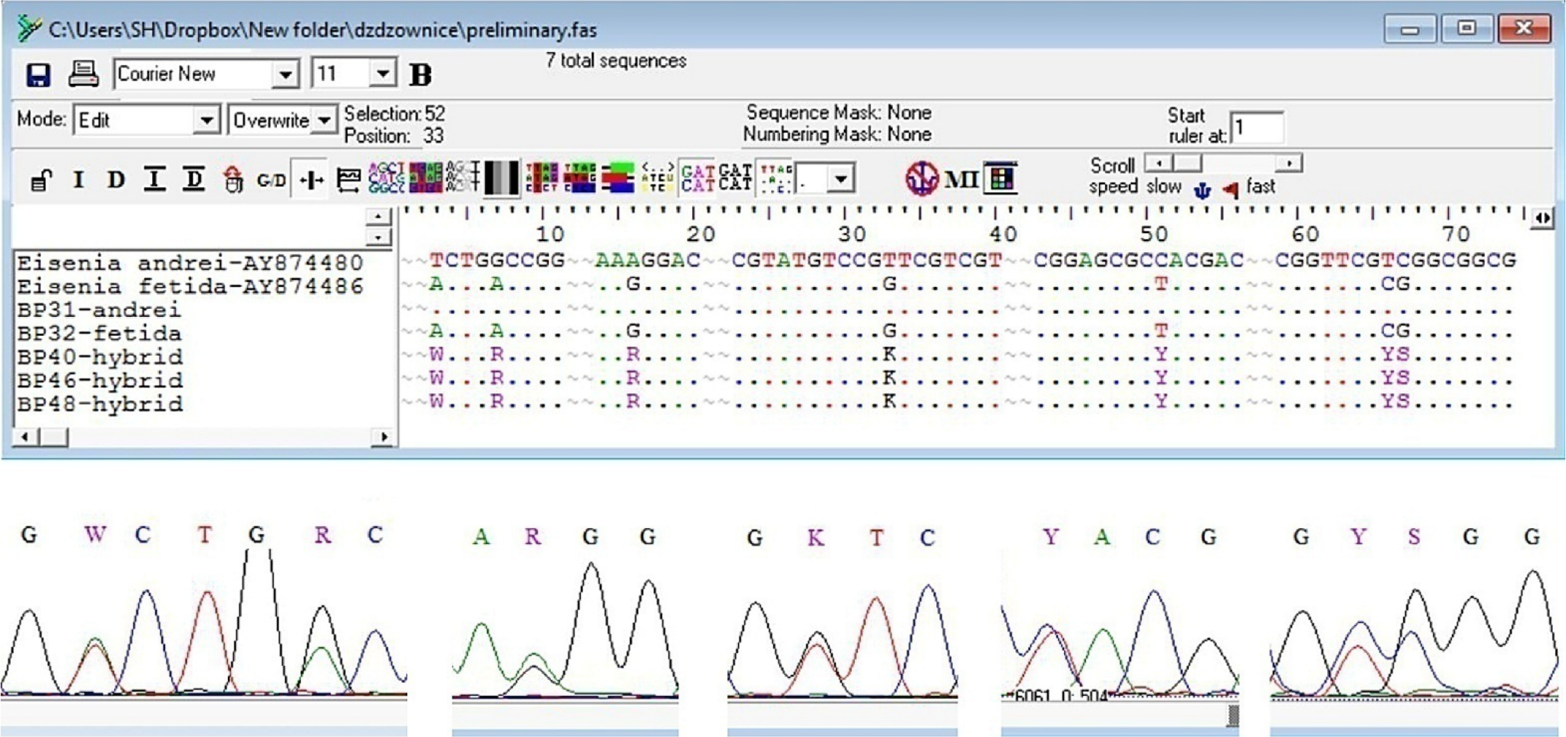
Species delimitation using molecular markers sequencing results. 28S sequences of *E*. *andrei* and *E*. *fetida* differ by 9 polymorphic sites, but only 7 of them were species specific and used for identification of hybrids.

A possibility of hybridization in couples of Ea and Ef was tested by analyzing species-specific sequences of the mitochondrial marker–cytochrome oxidase subunit I (COI) (‘a’ or ‘f’) showing maternity and the maternal/paternal nuclear marker 28S subunit of the nuclear ribosomal RNA (28S) (‘A’ or ‘F’). Ea and Ef are characterized by genotypes aAA and fFF, respectively. Hybrids of Ea+Ef possess two easily identified 28S sequences, AF/FA (Fig 2).

#### Labeling of investigated samples/specimens

Each investigated specimen was described by combining the mitochondrial and nuclear markers and its numerical code (reflecting its sequence in our analyses) as e.g. aAA45 (for Ea), fFF67 (for Ef), and aAF93 (for a hybrid from Ea ovum fertilized in Ea cocoon by Ef sperm), or fFA149 (for a hybrid from Ef ovum fertilized in Ef cocoon by Ea sperm). In a case of nuclear markers, either ‘AF’ or ‘FA’, on the first place there is the marker of maternal origin (i.e. the same origin as mitochondrial one). Amputation of easily regenerating posterior segments is not harmful for *Eisenia* specimens [30] and–if necessary—might be performed repeatedly; that was reflected by two or three numbers for several individuals, e.g. fFA149/194 or fFA143/159/191.

#### Phylogenetic relatedness

**Fig 3:**
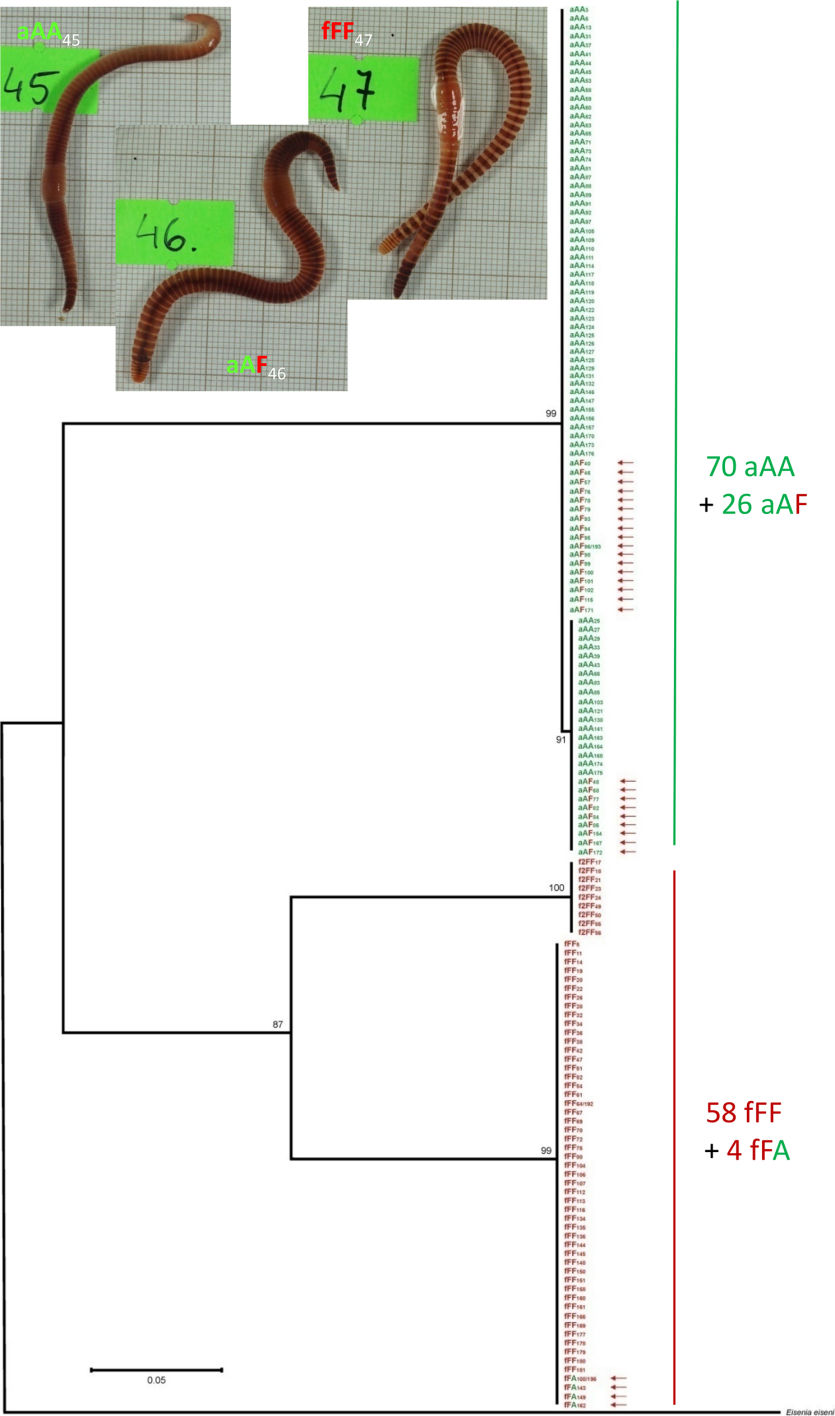
The maximum-likelihood phylogram for the COI gene of *Eisenia andrei/fetida* combined with the nucler 28S rRNA genes of the same individuals with the same code. Arrows indicate *E*. *andrei* x *E*. *fetida* hybrids. Bootstrap support is given. All sequences were deposited in the GenBank (MG030809—MG030998 for 28S and MG030999—MG031156 for COI). Inset: Phenotypes of *Eisenia andrei* (aAA45), *E*. *fetida* (fFF47) and the hybrid (aAF46). Regeneration blastemas of the posterior segments pointed out by the yellow arrows.

The phylogeny analyses indicated a high level of genetic differentiation between *E*. *andrei* and *E*. *fetida*. The p-distance between these two species were 0.150 for COI and 0.017 for 28S. For COI marker, intraspecies variation has also been found. The COI sequences for *E*. *fetida* formed two distinct subclades, ‘f’ and ‘f2’ separated with p-distance = 0.114. Two COI subclades were also present for *E*. *andrei*, but p-distance was negligible (Fig 3).

Fig 3 shows the maximum-likelihood phylogram for the mitochondrial COI gene of 158 specimens of *Eisenia fetida/andrei* combined with the nuclear 28S rRNA genes of the same coded individuals. This analysis showed three distinct clusters; one cluster of 96 individuals including exclusively mitochondrial ‘a’ COI sequences characteristic for *E*. *andrei* ova, but among them there are 70 ‘aAA’ sequences typical for *E*. *andrei* and 26 sequences ‘aAF’ characteristic for hybrids. The two remaining clusters consisted of 9 and 53 individuals from *E*. *fetida* ova, ‘f2’ and ‘f’, respectively, the former with only *E*. *fetida* nuclear sequences (‘f2FF’), and the later, with 49 ‘fFF’ and 4 ‘fFA’ sequences characteristic for hybrids.

### Earthworm phenotypes

The photos from the inset of Fig 3 demonstrate that among investigated earthworms there were specimens of uniformly red pigmentation typical for *E*. *andrei* (e.g. aAA_45_), specimens with striped red-yellow pigmentation patterns typical for *E*. *fetida* (e.g. fFF_47_), and the hybrids of intermediate pigmentation patterns (among them aAF_46_) with the slightly banded posterior segments. However, in several specimens the areas around the intersegmental grooves appearing either red or pale/yellow were not always as distinct as on the examples from Fig 3.

### Genealogy of hybrid descendants of *E*. *andrei* and *E*. *fetida* couples

The analysis of genotyped specimens from the controlled mating experiments allowed us to trace their genealogy. Pure aAA and fFF specimens were most common among 158 genotyped worms, thus only closest relatives of 30 hybrid earthworms (26 specimens from *E*. *andrei* ova, i.e. aAF, and 4 specimens from *E*. *fetida* ova, i.e. fFA) detected among descendants of the initial couples of *E*. *andrei* (aAA) with *E*. *fetida* (fFF) are presented in Table 2.

**Table 2:** Relationships between descendants of *E*. *andrei* (aAA) and *E*. *fetida* (fFF) couples with special attention to aAF (bold) and fFA (italic bold) hybrids genotyped for mitochondrial COI and nuclear 28S markers*.

Family No.		Parents	Offspring/generations
1	1a	aAA25 + fFF26	F1: [**aAF46**; aAA45]; [aAA62; fFF61]; **aAF57/90**; aAA58; aAA59; aAA63; aAA91; aAA92
	1b	**aAF46Mp** + aAA45	F2: aAA109; aAA110; **aAF95**; **aAF96/103**
	1c	aAA62 + fFF61	F1: [**aAF76/137**; aAA83/138]; [**aAF77/142**; aAA87]; [**aAF98/152**; aAA97/153]; aAA170; **aAF171**; **aAF172**; AA173;
	1d	**aAF76/137** + aAA83/138	F2: **aAF84/139**; aAA85/140; **aAF86/141**; aAA96; aAA141; aAA174; aAA175
	1e	**aAF77/142** + aAA87	F2: aAA88; aAA89
	1f	**aAF98/152 +** aAA97/153	F2: **aAF154**; aAA155; aAA156; aAA157; aAA176
2	2a	aAA37 + fFF38	F1: aAA65; fFF64/192; [**aAF78**; fFF75]
	2b	**aAF78 +** fFF75	F2: [**aAF79**; **aAF82**]
	2c	**aAF79** + **aAF82**	F3: ---
3	3a	aAA31 + fFF32	F1: aAA39; [aAA71; fFF72]; [**aAF99**; **aAF100**]; [**aAF101**; **aAF102**]
	3b	aAA71 +fFF72	F1: [**aAF93**; **aAF94**];
	3c	**aAF99** + **aAF100**	F2: ---
	3d	**aAF101** + **aAF102**	F2: ---
	3e	**aAF93** + **aAF94**	F2: ---
4	4a	aAA33 + fFF34	F1: **aAF40**; [fFF67; **aAF68**]; **aAF115**; fFF116
	4b	**aAF68** + fFF67	F2: fFF148; ***fFA149/194***; fFF150; fFF151; ***fFA162***; **aAF167**
	4c	***fFA149/194*** + **aAF86/141**	F3: ---
	4d	***fFA162*** + **aAF172**	F3: ---
5	5a	aAA29 + fFF30	F1: [**aAF48;** fFF47]
	5b	**aAF48 +** fFF47	F2: [***fFA108/196*;** fFF107/197]; [***fFA143/159/191***; fFF158]; fFF144; fFF145; fFF160; fFF161; fFF178; fFF179; fFF180; fFF181
	5c	***fFA108/196* +** fFF107/197	F3: ---
	5d	***fFA143/159/191*** + fFF158	F3: ---

*Symbols are the same as in Fig 3. Most of genotyped aAA and fFF specimens not closely connected with hybrids are neglected here. For brackets and underlined codes please see text.

In family 1, among progeny of the parents aAA25+fFF26 ten F1 specimens were genotyped, including seven Ea (aAA), one Ef (fFF), and two aAF hybrids (family 1a). The first pair of virgin specimens randomly taken from this family, identified at their adulthood as [aAA45+aAF46] formed the family 1b with two aAA specimens and two aAF hybrids among the F2 offspring. The second pair of virgin specimens randomly taken from the family 1a represented pure Ef and Ea genotypes [AA62+fFF61]. Therefore, although they belonged formally to F1 generation, we have marked them as parental generation and ten of their genotyped offspring as F1. Among them we identified five aAA and five aAF (family 1c). From them three backcrossed pairs of aAF hybrids with pure aAA earthworms were randomly generated. All of them giving viable F2 generation; i: [aAF76/137+aAA83/138] with two hybrids and five aAA offspring (family 1d); ii: [aAF77/142+ aAA87] giving two aAA offspring (family 1e) and iii: [aAA97/153 and aAF98/152] giving one hybrid and four aAA among F2 offspring (family 1f in Table 2).

In family 2, among four F1 specimens of the inter-specific parental pair (aAA37+fFF38), one aAA, two fFF specimens and one aAF hybrid were identified (family 2a). In the F2 generation obtained from the hybrid and fFF pairing [aAF78+fFF75], two hybrids aAF were observed (family 2b), while when F2 hybrids were intercrossed [aAF79+aAF82] cocoons but no hatchlings were noticed (family 2c) (Table 2).

In family 3, among F1 progeny of the aAA31+fFF32 couple there were two aAA specimens, one fFF, and four aAF hybrids (family 3a). Then, three pairs of F1 offspring were intercrossed. The [aAA71 + fFF72] pure species couple yielded two hybrids in F1 generation (family 3b), while intercrossing of hybrids from F1 generation of family 3a [aAF99+aAF100] and [aAF101+aAF102], or family 3b [aAF93+aAF94], gave only sterile cocoons by the end of investigations (families 3c,d,e in Table 2).

In family 4, the couple aAA33+fFF34 gave two fFF and three aAF hybrids among F1 offspring (family 4a). Intercrossing of F1 specimens [aAF68+fFF67] gave three Ef (fFF) specimens and two fFA hybrids in the F2 generation (family 4b). Each of the F2 hybrids demonstrating Ef specific COI mitochondrial markers, fFA149/194 and fFA162 (underlined in family 4b), was paired with the hybrid demonstrating COI characteristic for Ea, i.e. aAF86/141 (underlined in family 1d) and aAF172 (underlined in family 1c), respectively. Only sterile cocoons and no F3 hatchlings were present in their boxes [fFA149/194+aAF86/141] (family 4c), and [fFA162+aAF172] (family 4d) (Table 2).

In family 5, the pair aAA29 and fFF30 gave fFF47 and aAF48 in F1 offspring (family 5a). The F1 specimens [aAF48+fFF47] were intercrossed and gave plenty of genotyped offspring in F2 generation, among them ten fFFs and two fFA hybrids (family 5b). From them, the fFA108/196 and fFA143/159/191hybrids were intercrossed with fFF specimens from the F2 generation ([fFA108/196+fFF107/197] and [fFA143/159/191+fFF158]) giving plenty of cocoons but no F3 hatchlings till the end of present investigations (families 5c and 5d in Table 2).

In general, among 26 aAF hybrids detected during present investigations, the majority (18 specimens) derived from inter-specific couples (aAA+fFF), while 8 of the remaining aAF hybrids were offspring of the backcrosses, either (aAF+aAA) or (aAF+fFF). In contrast, no fFA hybrids were observed in F1 generations from aAA+fFF inter-specific pairs. All four of fFA hybrids derived in F2 generation from the back-crosses of the F1 aAF hybrids with fFF specimens.

Despite cocoon production, offspring was absent in hybrid-hybrid pairs, both aAF+aAF or aAF+fFA. So far, the back-crosses of fFA with fFF specimens have not given viable offspring in F3 generation (Table 2).

#### Speculations on possibilities of self-fertilization, cross-fertilization, and hybridization in mating pairs used in present studies

**Fig 4:**
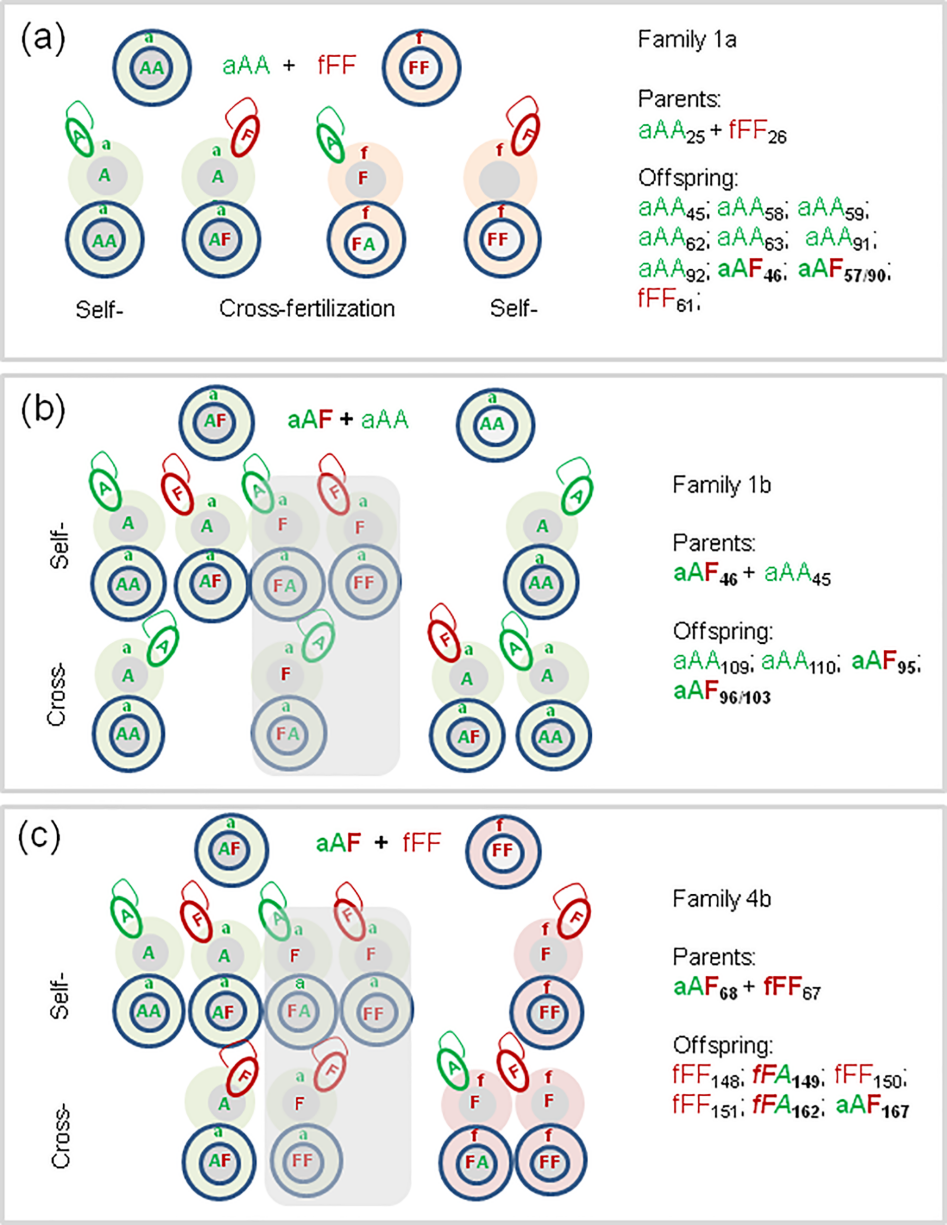
Speculations on inheritance of mitochondrial (‘a’, ‘f’) and nuclear (‘A’, ‘F’) markers by self- and cross-fertilization of ‘aA’ and ‘fF’ ova of aAA and fFF parental species, and aAF hybrids with each of parental species. a) crosses of pure species; b) back-cross of (aAF+aAA) couple; c) back-cross of (aAF+fFF) couple. Parts of Fig 4B and 4C with incompatible ‘aF’ova are shadowed as less probable. The examples were taken from Table 2, families 1a, 1b, and 4b.

As illustrated on Fig 4A, the progeny of an inter-specific couple Ea+Ef may contain aAA and fFF earthworms resulted from self-fertilization of ‘aA’ or ‘fF’ ova by spermatozoa of the same specimens. Inter-specific hybrids aAF resulted from cross-fertilization of ‘aA’ ova by ‘F’ spermatozoa of fFF parent, and vice versa, fFA hybrids from ‘fF’ ova fertilized by ‘A’ spermatozoa. Among progeny of inter-specific couples investigated in the present work we did find the aAA, fFF, and aAF earthworms while fFA hybrids were absent (exemplified by family 1a from Table 2).

The aAF hybrids obtained in the present studies gave vital and fertile offspring in backcrosses both with aAA and fFF specimens (Fig 4B and 4C, respectively).

The hybrids shall produce two types of spermatozoa, with either ‘A’ or ‘F’ nuclear markers, and also two types of ova, being either compatible with respect of mitochondrial and nuclear markers, i.e. ‘aA’ or ‘fF’, or incompatible with respect of these markers, like ‘aF’or ‘fA’. There are findings showing that the latter possibility is less probable in several species [33, 34], thus for simplicity this was excluded from further speculations (shadowed on Fig 4B and 4C).

Fig 4B showed that the two hybrids (aAF95 and aAF96/103), being the offspring of (aAF46+ aAA45) couple, may be resulted not only from self-fertilization of ‘aA’ ovum of the aAF46 hybrid with the ‘F’ spermatozoon of the same earthworm, but also by cross-fertilization of ‘aA’ ovum of the aAA parent with the ‘F’ spermatozoon of the aAF46 hybrid. The aAA109 and aAA110 earthworms might be descended either from self- or cross-fertilization of the ‘aA’ ova of the aAF hybrid, or self- or cross-fertilization of the ‘aA’ ova of the aAA earthworm by the ‘A’ spermatozoa of one of these partners.

Similar possibilities exist in the case of the aAF back-cross with the fFF partner (Fig 4C). Both fFA149 and fFA162 hybrid offspring had to result from fertilization of ‘fF’ ova of fFF67 partner by the ‘A’ spermatozoa of the aAF68. However, aAF167 hybrid might have resulted from fertilization of the ‘aA’ ova either by the ‘F’ spermatozoon of the same aAF68 hybrid specimen or by the cross-fertilization of the ‘aA’ ovum of the hybrid with the ‘F’ spermatozoon of the FF67 partner. Also fFF148, fFF150, and fFF151 offspring might have resulted from either self- or cross-fertilization of ‘fF’ ova of the fFF67worm (Fig 4C).

In conclusion, in the case of aAF hybrids being the F1 offspring of Ea+Ef pair, the ‘aA’ ovum of aAA partner had to be fertilized by the ‘F’ spermatozoon from the fFF partner. However, analysis of COI and 28S genes performed in the present investigations was not sufficient for precise identification of parents of aAF hybrids in the progeny of backcrosses of aAF with aAA or fFF worms (Fig 4). In such a case studies of highly polymorphic microsatellite loci [35–37] shall give conclusive explanation. E.g. this method was sensitive enough to analyze multiple paternity in *Hormogaster elisae*, when one mother produced descendants from more than one partner [38, 39].

## Discussion

The better reproductive performance of *E*. *andrei* than *E*. *fetida* demonstrated in earthworms from the present experiment was also described by other scientists who investigated these species derived from other localities [18, 19, 40]. Earthworm reproductive performance changes in annual cycle, e.g. maximum mating activity of the field population of *E*. *fetida* was achieved in spring [41]. The drastic differences in percentages of sterile cocoons between *E*. *andrei* and *E*. *fetida* (7% and 48%, respectively) from present experiments might reflect different reproduction dynamics of these species that, hypothetically, might be affected by the actual length of their laboratory ‘domestication’. Bimodal reproduction with spring and autumn maxima of cocoon laying/hatching and high sterility rate of inter-seasonally laid cocoons was described in another species, *Eisenia lucens* [42].

Reproductive performance of the laboratory-formed inter-specific Ea+Ef pairs were investigated in present studies on earthworms from France, by [18] on earthworms from South Africa and France, and by [19] on earthworms from Spain and Brasil. The results of all these studies revealed extensive cocoon production by Ea+Ef pairs. However, only the inter-specific pairs of earthworms from France produced viable cocoons and hatchlings. Future experiments shall address comparative studies on hybridization of earthworms cultured in various laboratories and/or from various natural populations.

### Presence of hybrids between *E*.*andrei* and *E*. *fetida*

The earthworms from *Eisenia* sp. investigated during present studies formed three distinct clusters on phylogenetic tree based of haploid mitochondrial COI genes of maternal origin, one including exclusively ‘a’ sequences of *E*. *andrei*, and two including ‘f’ and ‘f2’ sequences of *E*. *fetida*. These results fit perfectly to data obtained by the international group of experts EBI (‘*Eisenia* Barcoding Initiative’, EBI) who analyzed mitochondrial COI sequences of the coded fixed specimens provided from 28 laboratories from 15 countries [16]. This analysis revealed three distinct haplotype clusters; one including exclusively *E*. *andrei* sequences, and two with *E*.*fetida* sequences, the latter being hypothetically two different cryptic species, *E*. *fetida 1* and *E*. *fetida 2*. Two very divergent haplotypes of *E*. *fetida* COI sequences were detected also during other studies [13, 14, 30], thus this aspect is worth of further investigation. Remarkably, specimens of the molecular *E*. *fetida* clusters from EBI studies based on COI genes were always identified morphologically as *E*. *fetida*. However, this was not true the other way round, i.e., some specimens of the molecular *E*. *andrei* cluster were identified morphologically as *E*. *fetida* by the EBI group [16]. However, our results suggest that at least some of *E*. *fetida*-like specimens among *E*. *andrei* cluster might be rather interspecific hybrids ‘aAF’.

Recently it turned out that the COI cluster of *E*. *andrei* also contains six distinct lineages, irrespective of the striped or uniform red coloration [17]. Hypothetically, such a discordance between coloration and molecular delimitation based on COI genes might be at least partly connected with hybridization in the natural field populations of *E*. *andrei* with *E*. *fetida*.

The novelty of the present studies is that each earthworm from three clusters of phylogenetic tree based on COI gene sequences of maternal origin is characterized also by its diploid maternal/paternal 28S genes, being either ‘AA’ or ‘FF’ for *E*. *andrei* and *E*. *fetida*, respectively, or ‘AF/FA’ for hybrids. It turned out that within the *E*. *andrei* COI-clade there are 70 aAA earthworms of *E*. *andrei* species and 26 of interspecific aAF hybrids. One of the two *E*. *fetida* COI-classed contains exclusively nine fFF specimens, but within the second COI-clade there are 49 fFF *E*. *fetida* specimens and four fFA hybrids. For the first time these results give a formal proof of the existence of viable interspecific hybrids between *E*. *andrei* and *E*. *fetida*. As reviewed by [19], the existence of such hybrids was suggested by some investigators but negated by others.

Sheppard [22] found hatchlings in crosses between *E*. *andrei* and *E*. *fetida* and stated that the offspring could be the result of hybridization, self-insemination or ‘facilitated self-fertilization’ connected with sperm mixing. The results of our present experiments and molecular analyses fully confirmed his suppositions as we have proved that among F1 offspring of aAA and fFF pairs there are both the earthworms of the pure species (aAA and fFF) derived from self-fertilization and the hybrids aAF derived from cross-fertilization. Self-fertilization of individually cultured virgin specimens of *E*. *andrei* and *E*.*fetida* was recorded as being quite common in earthworms from Spain [29] but such phenomenon is very seldom in *Eisenia* sp. from France (in progress). For this reason we assume that appearance of aAA or fFF offspring of (Ea+Ef) pairs is a result of self-fertilization facilitated by the partner from closely related species by mixing sperm of the both copulating earthworms. Other studies will be addressed to quantify incidences of facilitated self-fertilization and hybridization in (Ea+Ef) pairs.

### Hybrid fertility and evolutionary implications

**Fig 5:**
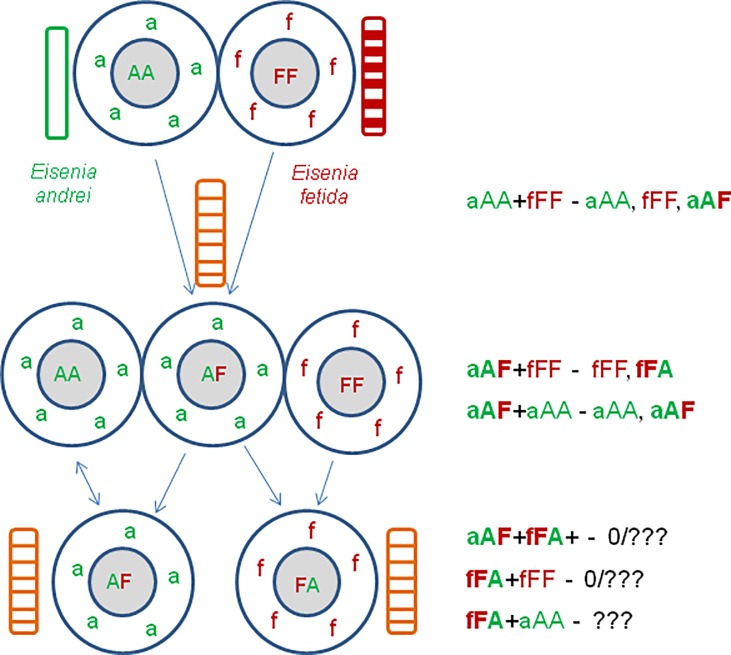
Summary of the results of experiments on the controlled laboratory mating of *E*. *andrei* with *E*. *fetida*, and their hybrids. Flow of genes responsible for pigmentation patterns.

The next question addressed in the present studies was whether interspecific hybrids are fertile. To answer this question, the virgin hybrid worms were mated with other hybrids or back-crossed with each of the parental species. The results obtained so far evidenced clearly that at least aAF hybrids are fertile and gave progeny with the proper partner (Fig 5).

In present experiments, among offspring of Ea+Ef pairs there were aAA and fFF worms and aAF hybrids but none fFA hybrids. The latter appeared among offspring of backcrosses of aAF hybrids with fFF earthworms. The aAF hybrids gave also progeny (aAA and aAF) in backcrosses with aAA earthworms. Despite of normal cocoon production, offspring was absent so far in hybrid-hybrid pairs and in the back-crosses of fFA with fFF specimens. Among hybrids, there are earthworms of various pigmentation patterns resulted from introgression of genes from distinctively striped *E*. *fetida* to uniformly pigmented *E*. *andrei* (Fig 5, see photos on Fig 3).

The results obtained so far suggest that EaxEf hybridization is asymmetrical. The aAF hybrids from Ea ova are relatively frequent, and they can breed with both Ea and Ef specimens, giving next generations of aAF hybrids and their progeny or serving as donor of spermatozoa to fFF partners resulting in fFA hybrids. However, the latter type of hybrids were very sparse and without offspring till the end of present investigations (Fig 5). Asymmetrical hybridization is known in other species, e.g. like that between *Operophtera brumata* and *O*. *bruceata* (Lepidoptera: Geometridae) from the United States [43].

Hypothetically, the more common aAF hybrids descended from the aA ova of aAA earthworms. This might be connected with better reproductive performance of *E*. *andrei* than *E*. *fetida*, recorded not only in earthworms from France used in present experiments but also in earthworms from Spain and Brasil investigated by other scientists [18, 19, 40]. However, dynamic of reproduction can be altered with moisture/temperature/food source [40; 41, 42]. Therefore it is possible that various factors like seasonal cyclic changes and environmental contaminants might have different effects on the two species, including influence on their hybridization abilities. Such phenomenon is known in other species, e.g. in the temporally varying hybridization of the North Atlantic eels *Anguilla anguilla* and *A*. *rostrata* [44] and in temperature-dependent hybridization of *Drosophila melanogaster* and *D*. *stimulans* [45].

All the *E*. *fetida* parents of all hybrid earthworms from present experiments, both aAF and fFA, have derived from the ‘f’ lineage of *E*. *fetida/andrei* phylogenetic tree, with no contribution from the ‘f2’ lineage. Studies on the reproductive performance of laboratory pairing of fFF and f2FF earthworms might also be revealing as to whether they are two cryptic sibling species. It is an object of research in progress.

## Conclusions

Laboratory mating of virgin earthworms Ea+Ef gave both self-fertilized specimens of aAA and fFF genotypes, and some aAF hybrids derived from aA ova of Ea. These hybrids mated with the parental species gave new generation of aAA and aAF hybrids and so far sterile fFA hybrids from fF ova of the Ef partner. Pairs of two hybrids produced plenty sterile cocoons. Hypothetically, such asymmetric hybridization may be modulated by several external factors, a phenomenon worth of further inquiry.

The existence of Ea and Ef hybridization make these common species easily maintained in laboratory the attractive models for studies on mechanisms of interspecies gene flow during introgressive hybridization [46, 47], phenomenon described in other lumbricids, *Allolobophora* sp. [48] and *Lumbricus* sp. [49].
